# Matters arising: a commentary on “Failing to succeed: advancing mechanistic understanding of implementation strategies through retrospective and prospective use of causal pathway diagrams” by Austad et al. 2025

**DOI:** 10.1186/s43058-026-00914-1

**Published:** 2026-04-21

**Authors:** Bo Kim, Cara C. Lewis

**Affiliations:** 1 Center for Health Optimization and Implementation Research, VA Boston Healthcare System, Boston, MA USA; 2 Department of Psychiatry, Harvard Medical School, Boston, MA USA; 3 Center for Translation Research and Implementation Science, National Heart, Lung, and Blood Institute, National Institutes of Health, Bethesda, MD USA

## Progress and challenges in studying implementation mechanisms

Implementation science is rapidly evolving to increase its explanatory capacity. Early work focused on answering the question, “What went wrong?” by characterizing barriers to evidence-based intervention (EBI) uptake. Subsequent efforts began to address the question, “What can we do?” by offering ontologies for classifying implementation strategies intended to overcome those barriers. Most recently, the field has turned toward an arguably more complex question: how do specific strategies work, under what circumstances, to overcome which barriers and produce which outcomes [1]? This shift reflects growing recognition that testing bundles of strategies against distal outcomes, without attention to underlying causal processes, limits cumulative learning and generalizability (post hoc). Moreover, without mechanistic specification, strategy bundles risk becoming overly complex and misaligned with contextual constraints, as teams may add components without clear justification for how each is expected to activate specific and unique mechanisms (a priori).

Austad et al. offer a prime example of how mechanistic inquiry conveys practical and scientific benefits to the inherent complexity of implementation research [2]. Their study brings value beyond the particulars of metabolic-associated steatotic liver disease screening, as the case study makes concrete how mechanistic reasoning can be applied a priori and post hoc in implementation studies. By foregrounding causal pathway diagrams (CPDs) as a tool for centering implementation mechanisms [3], particularly when strategies fail, the paper offers an opportunity to reflect on where the field is making progress and where conceptual and methodological challenges remain.

## Failure as a productive entry point for mechanistic inquiry

A central contribution of the paper is its use of CPDs retrospectively, as a tool for diagnosing implementation failure. This orientation is consequential. Rather than assuming intact causal chains simply underperform, retrospective CPDs make visible where mechanisms fail to activate, where preconditions are unmet, or where moderators attenuate effects.

This approach advances mechanistic inquiry beyond descriptive post-hoc explanation. Failure is treated not as noise or execution error, but as analytically productive – an opportunity to interrogate causal assumptions embedded in strategy design [4]. In doing so, the paper aligns with a broader “fail forward” orientation emerging in implementation science, one that treats failure as a necessary input for theory refinement rather than an endpoint to be avoided or explained away.

This orientation resonates with broader efforts to design and evaluate complex interventions with mechanisms in mind and to understand why implementation trials succeed or fail. For example, work by Dixon-Woods and colleagues has emphasized examining trial failures as opportunities to interrogate assumptions embedded in design and delivery [5], while Kislov and colleagues’ theorizing underscores the importance of explicitly linking mechanisms to multilevel contextual conditions [6]. Situating the study of mechanisms within this wider landscape reinforces their value and presents a range of opportunities for approaching this work.

Importantly, some of the challenges identified retrospectively in the Austad et al. case might plausibly have been anticipated a priori. This observation underscores a value proposition for making mechanism articulation easier to do within and more routinely conducted by implementation research teams. This does not diminish the value of retrospective CPD use; rather, it underscores the iterative nature of mechanistic inquiry. When mechanisms are treated as situated and context-dependent rather than fixed properties of strategies, prospective specification and retrospective refinement become complementary activities. Failure analysis thus contributes not only to explanation after the fact, but to strengthening prospective design in subsequent iterations [7].

## Advancing precision: mechanisms, moderators, preconditions, and proximal outcomes

The paper also advances conceptual precision, in practically meaningful ways, by explicitly distinguishing among determinants, mechanisms, moderators, preconditions, and proximal outcomes [8]. These elements are frequently conflated in mechanistic accounts, leading to ambiguity about what actually produces change, what conditions enable change, and what influences its magnitude.

CPDs require these distinctions to be made explicit. In doing so, they reveal both the analytic gains and the empirical challenges of achieving such precision. As the case illustrates, boundaries among these constructs are not always stable across contexts or implementation phases. Yet the effort to specify them remains conceptually and practically valuable precisely because it surfaces uncertainty that clarifies decision points, rather than obscuring uncertainty lost to inertia.

This tension reflects a broader challenge for the field: how to sharpen mechanistic specification without lapsing into false certainty. This tension also points to a need for more explicit training in mechanistic inquiry within implementation science programs. Developing facility with distinguishing mechanisms from moderators, preconditions, and proximal outcomes – and with treating mechanistic models as provisional rather than definitive – requires methodological skills that are not systematically taught. Without such training, efforts to advance mechanistic precision risk oversimplification or overconfidence, undermining the cumulative learning these approaches are intended to support. In this sense, the relational clarity achieved through CPDs reflects a broader methodological principle in mechanistic inquiry: precision in specifying how elements connect is more consequential than the particular diagrammatic or analytic form used to represent them.

The paper demonstrates that CPDs can support this balance by functioning as provisional causal hypotheses, or micro-theories, that are open to revision as empirical evidence accumulates, thus advancing the science of implementation. Importantly, Austad et al. uncover how CPDs yield practical benefits with the explicit identification of proximal outcomes, which provide opportunities for early signal testing that are more responsive to and respectful of implementation partners. By attending to proximal outcomes, teams can assess whether strategies are activating intended mechanisms in near real time, rather than waiting months or years for distal indicators such as EBI fidelity or clinical outcomes. This shift enables rapid modification of strategies when signals suggest misalignment or attenuation, reducing the burden on interest holders, and potentially avoiding implementation failures. In this sense, proximal outcomes function not only as analytic markers, but as practical indicators for adaptive, partner-centered implementation.

Although this paper focuses on CPDs, the broader insight extends beyond this particular method. Across approaches such as directed acyclic graphs, mechanism mapping, and other relational modeling techniques, the core contribution lies in making explicit the relationships among determinants, mechanisms, preconditions, moderators, and proximal outcomes. What is generalizable across these approaches is not the labeling of constructs per se, but the disciplined and precise articulation of how they are hypothesized to relate to one another.

## Mechanisms as situated, negotiated, and informed by multiple forms of knowledge

A particularly important contribution of the paper – and one that extends current mechanistic discussions – is its implicit attention to whose knowledge informs mechanistic explanation. Mechanisms are not purely technical constructs; they are isolated, labeled, and operationalized through user perspectives. Accordingly, knowledge exists in multiple forms, including empirical research evidence, theoretical underpinnings, clinical and operational expertise, and the experiential and contextual knowledge of those involved in implementation.

Interest holder engagement is not ancillary to mechanistic validity but central to it [9]. They hold forms of experiential and contextual knowledge that are often unavailable through formal data sources alone. Without the descriptive, nuanced, lived experience of those embedded in implementation contexts, researchers risk mischaracterizing barriers and, in turn, targeting misaligned mechanisms through strategy selection and design. This perspective aligns with community-engaged approaches where implementation strategies are designed within community-defined priorities and constraints [10].

Community-engaged CPD development may center outcomes such as reducing disparities, which likely surface structural and systemic barriers that may otherwise remain implicit or outside of the purview of implementation design. By making these determinants explicit, CPDs require articulation of mechanisms that show promise of activation through strategies that may not typically be selected when implementation outcomes are defined in isolation and strategies are built in bundles of those that are familiar and feasible. Few published examples illustrate how CPDs have been used in this way, highlighting an important opportunity for future mechanism-focused research.

## From generalizability to mechanistic transportability

Critiques of implementation methods often hinge on questions of generalizability [11]. The paper invites a productive reframing: rather than asking whether CPDs generalize, we might ask what aspects of CPDs travel across contexts.

Seen in this light, CPDs may contribute most by identifying classes of mechanisms and common failure modes, rather than by offering fixed or universally transportable causal pathways. This distinction is important. It shifts attention from replication of diagrams to transfer of mechanistic insight, which supports adaptation while avoiding over-standardization. Moreover, CPDs as micro-theories should be refined with empirical investigations to explore which aspects are robust across contexts.

This framing positions CPDs as complementary to other mechanistic approaches, such as mechanism mapping [12] or realist synthesis [13], rather than as a unifying solution. Each approach brings different assumptions, inputs, analytic affordances, outputs, and functions, and their combined use may be more generative for the field than privileging any single method.

## Implications for the next phase of implementation mechanisms research

The paper highlights several priorities for the next phase of mechanisms research in implementation science. First, mechanistic hypotheses generated through CPDs require prospective testing. Retrospective insight, used alone, risks remaining explanatory rather than predictive.

Second, advancing implementation theory will require intentional integration of complementary mechanistic methods [14, 15], recognizing that different approaches serve distinct purposes, require different inputs and expertise, and generate different forms of insight. Taken together, their outputs can enrich the field’s capacity to anticipate strategy impacts in diverse contexts.

Finally, the paper surfaces the importance of theory building. CPDs actively engage teams in theorizing, but they are most robust when grounded in established disciplinary theories of behavior and system change, or when they build theories for multilevel implementation. As CPDs are tested across contexts, their function as micro-theories can contribute upward to mid-range and grand theory development. To date, this opportunity remains under-realized.

## CPDs as a practice for asking better mechanistic questions

Each CPD use case offers a practical reference point for others working with similar strategies, EBIs, or contexts. Yet the contribution of CPDs may extend beyond their visual outputs. Prospective CPD use demands a balance of precision and parsimony: selecting discrete strategies, isolating their mechanisms of action, assessing whether preconditions can be met, operationalizing moderators, and then connecting discrete strategy CPDs to assemble strategy bundles aligned with partner outcomes and goals.

CPDs may thus serve as a tool to ask more sophisticated mechanistic questions. By demanding explicit causal reasoning at each step of strategy selection and development, they strengthen the field’s capacity for disciplined, theory-informed, cumulative learning – an essential foundation for mechanistic maturity in implementation science.

## Data Availability

No datasets were generated or analysed during the current study.

## Abbreviations

CPD: Causal pathway diagram
EBI: Evidence-based intervention
