# Correction: Matters arising: a commentary on “Failing to succeed: advancing mechanistic understanding of implementation strategies through retrospective and prospective use of causal pathway diagrams” by Austad et al. 2025

**DOI:** 10.1186/s43058-026-01018-6

**Published:** 2026-06-23

**Authors:** Bo Kim, Cara C. Lewis

**Affiliations:** 1 Center for Health Optimization and Implementation Research, VA Boston Healthcare System, Boston, MA USA; 2 Department of Psychiatry, Harvard Medical School, Boston, MA USA; 3 Center for Translation Research and Implementation Science, National Heart, Lung, and Blood Institute, National Institutes of Health, Bethesda, MD USA

**Correction: Implementation Science Communications (2026) 7:79**

10.1186/s43058-026-00914-1

In this article, the Competing interests statement was incorrectly published as:  Dr. Lewis receives royalties from Springer Publishing. The correct statement is:  Dr. Lewis receives royalties from Springer Publishing.  Dr. Kim is an Associate Editor for Implementation Science Communications.

The original article has been corrected.

